# (*R*,*S*)-Tetrahydropapaveroline production by stepwise fermentation using engineered *Escherichia coli*

**DOI:** 10.1038/srep06695

**Published:** 2014-10-21

**Authors:** Akira Nakagawa, Chiaki Matsuzaki, Eitaro Matsumura, Takashi Koyanagi, Takane Katayama, Kenji Yamamoto, Fumihiko Sato, Hidehiko Kumagai, Hiromichi Minami

**Affiliations:** 1 Research Institute for Bioresources and Biotechnology, Ishikawa Prefectural University, Nonoichi-shi, Ishikawa 921-8836, Japan; 2 Division of Integrated Life Science, Graduate School of Biostudies, Kyoto University, Oiwake-cho, Kitashirakawa, Sakyo-ku, Kyoto 606-8502, Japan

## Abstract

Tetrahydropapaveroline (THP), a benzylisoquinoline alkaloid (BIA) found in diverse pharmaceutical compounds, is used as a starting material for the production of BIA. THP also has various neurobiological properties but is difficult to synthesize. Therefore, a simple method for THP production is desired. Recent studies have shown that microbes, especially bacteria, can serve as platforms for synthesizing these complex compounds; however, because bacteria lack organelles, the designed synthetic pathway cannot be compartmentalized. Thus, the metabolic flow is frequently inhibited or disrupted by undesirable reactions. Indeed, in the first attempt to synthesize THP using a single strain of engineered *Escherichia coli*, the yield was quite low (<5 μM), mainly because of the oxidation of THP by tyrosinase, an essential enzyme in our production system. To circumvent these problems, we constructed a stepwise (*R*,*S*)-THP production system, in which the dopamine-producing step and the subsequent THP-producing step were separated. The yield of (*R*,*S*)-THP reached 1.0 mM (287 mg/L), the highest yielding BIA production method using a microbe reported to date. Furthermore, we demonstrated that (*R*,*S*)-THP produced by stepwise fermentation is useful for the production of reticuline, an important BIAs intermediate. Based on these observations, applying the stepwise fermentation method is discussed.

Benzylisoquinoline alkaloids (BIAs) include many pharmaceutical compounds, such as berberine (antidiarrheal), sanguinarine (antibacterial), morphine (analgesic) and codeine (antitussive) (Fig. S1). Because of requirement for complex reactions, it has been difficult to construct practical methods for BIA production using traditional synthetic methods, and pharmaceutical BIAs are primarily obtained by extraction from plants, despite their low content. Recently, microbial production of BIA has been reported as an alternative method to plant extraction^1^,^2^,^3^,^4^. Using microbes enables the production of high yields of BIA in a short time with low costs^3^.

THP possesses the basic skeleton of BIA, from which various BIAs can be obtained by through modifications such as methylation and C-C bond formation. Indeed, THP was used as a starting material for the microbial production of BIA^2^,^4^. THP is naturally produced by the condensation reaction of dopamine and 3,4-dyhydroxyphenylacetaldehyde (3,4-DHPAA), which is synthesized from dopamine by an endogenous monoamine oxidase (MAO) in humans. THP, extensively studied since the 1960's^5^, has various neurobiological properties, including reduction of dopamine^6^,^7^ and serotonin synthesis^8^, decreasing dopamine uptake^9^, and induction of necrotic and apoptotic cell death via oxidative stress^10^,^11^,^12^. THP is found in the urine of parkinsonian patients medicated with L-DOPA^13^,^14^, and the brain of rats administered L-DOPA^15^; furthermore, the presence of THP is related to the suppression of cocaine seeking behavior in rats^7^. Thus, THP is also important for neurobiology studies.

Although THP is in high demand for BIA production and neuronal research, it is difficult and expensive to obtain. Therefore, a simple method for THP production is desired. The biotransformation of dopamine to THP was performed by using *Aspergillus niger* (*A. niger*), which expresses native MAO. However, this system required dopamine as a substrate, and the yield was low (0.03 mM)^16^.

We report the construction of a (*R*,*S*)-THP production system by altering the reticuline synthetic pathway^3^, previously constructed using *Escherichia coli*. First, glycerol is converted to tyrosine via a tyrosine over-producing pathway, and then is oxidized to L-DOPA by tyrosinase (TYR) (Fig. 1). L-DOPA is catalyzed to dopamine by DOPA decarboxylase (DODC) and MAO oxidizes dopamine to 3,4-DHPAA. Dopamine and 3,4-DHPAA are finally condensed to (*R*,*S*)-THP via the non-enzymatic Pictet-Spengler reaction.

Because THP is a relatively unstable and reactive BIA because of its two catechol moieties, stabilization of THP is important for (*R*,*S*)-THP production. The tyrosinase used in the BIA production pathway is a copper containing enzyme with both tyrosine hydroxylase and *o*-diphenolase activities. Whereas tyrosine hydroxylase activity is essential for L-DOPA synthesis in the microbial production system of BIA (Fig. 1), *o*-diphenolase activity is undesirable because it results in the oxidation of the hydroxyl groups of the intermediate compounds, L-DOPA and dopamine, to their quinone derivatives^17^. In addition to these two compounds, we speculated that THP was also oxidized by the *o*-diphenolase activity of tyrosinase.

To establish an easy method for supplying THP, which is an important molecule in BIA production and neurobiological research, we attempted to construct a microbial production system. Herein, we report that THP is oxidized by tyrosinase and to avoid oxidation, we employed stepwise fermentative production of (*R*,*S*)-THP by using dopamine production and MAO expression strains.

## Results

### (*R,S*)-THP production using a single culture

We first attempted to produce (*R*,*S*)-THP using a single strain (AN1428) which contains the complete machinery to synthesize (*R*,*S*)-THP. However, only trace levels (less than 5 μM) of (*R*,*S*)-THP was produced by AN1428. In a previous study, we found that THP was quite unstable in the BIA production culture^3^. Taken together with the fact that THP has two catechol moieties, we speculated that the low THP productivity of AN1428 was as a result of the *o*-diphenolase activity of tyrosinase. Thus, we measured the *o*-diphenolase activity toward THP by using purified recombinant tyrosinase. As expected, THP was oxidized by tyrosinase, and the oxidation ratio of THP was about 69% compared with that of L-DOPA, a value that was approximately four times higher than that of dopamine (Table 1). These data indicated that THP was quite unstable in the culture of the tyrosinase expressing strain.

### Stepwise culture strategy for (*R,S*)-THP production

**Figure 1:**
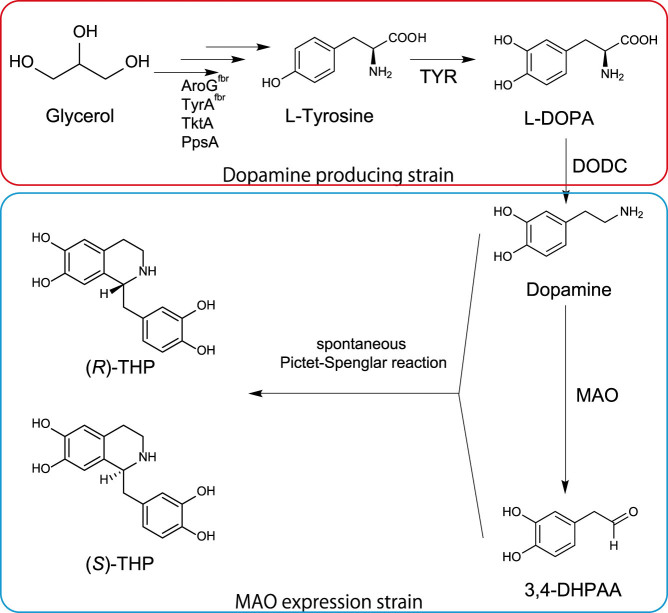
(*R*,*S*)-THP synthetic pathway constructed in *E. coli* strains. The synthetic pathway from glycerol to 3,4-DHPAA has been previously reported^3^. In the previous system, norcoclaurine synthase (NCS) was used for THP synthesis. However, this system was constructed without NCS as described in the Discussion. The (*R*,*S*)-THP synthetic pathway employed two strains, dopamine production strain (red blanket) and monoamine oxidase expression strain (blue blanket). AroG^fbr^, feedback-inhibition-resistant (fbr) 3-deoxy-D-arabino-heptulosonate-7-phosphate synthase; TyrA^fbr^, fbr-chorismate mutase/prephenate dehydrogenase; PpsA, phosphoenolpyruvate synthetase; TktA, transketolase; TYR, tyrosinase of *Ralstonia solanacearum*; DODC, DOPA decarboxylase of *Pseudomonas putida*; MAO, monoamine oxidase of *Micrococcus luteus*.

The produced THP would be oxidized by tyrosinase, which is an essential enzyme for THP synthesis. Thus, it would be difficult to develop the (*R*,*S*)-THP production system using a single strain. For efficient (*R*,*S*)-THP production, the final step of THP synthesis (condensation of dopamine and 3,4-DHPAA, Fig. 1) should be separated from L-DOPA production which is catalyzed by tyrosinase. There are two ways of separating this pathway, between L-DOPA production and (*R*,*S*)-THP production from L-DOPA, and between dopamine production and (*R*,*S*)-THP from dopamine. If the pathway was separated at the step of L-DOPA production, L-DOPA would be degraded by the *o*-diphenolase activity of tyrosinase. However, because the *o*-diphenolase activity toward dopamine was much lower than that toward L-DOPA (Table 1), dopamine should be relatively stable in the culture of the tyrosinase expression strain. Hence, we chose to produce THP via dopamine production from a simple carbon source and (*R*,*S*)-THP conversion from dopamine by using two strains and a two-step culture. First, dopamine was produced from a simple carbon source using the dopamine producing strain (AN1126), and then this strain was eliminated by centrifugation to prevent contamination of the second step culture. For the second step, the harvested supernatant containing dopamine was mixed with the MAO expression strain (AN1055) for (*R*,*S*)-THP production.

### Fermentative production of dopamine

**Figure 2:**
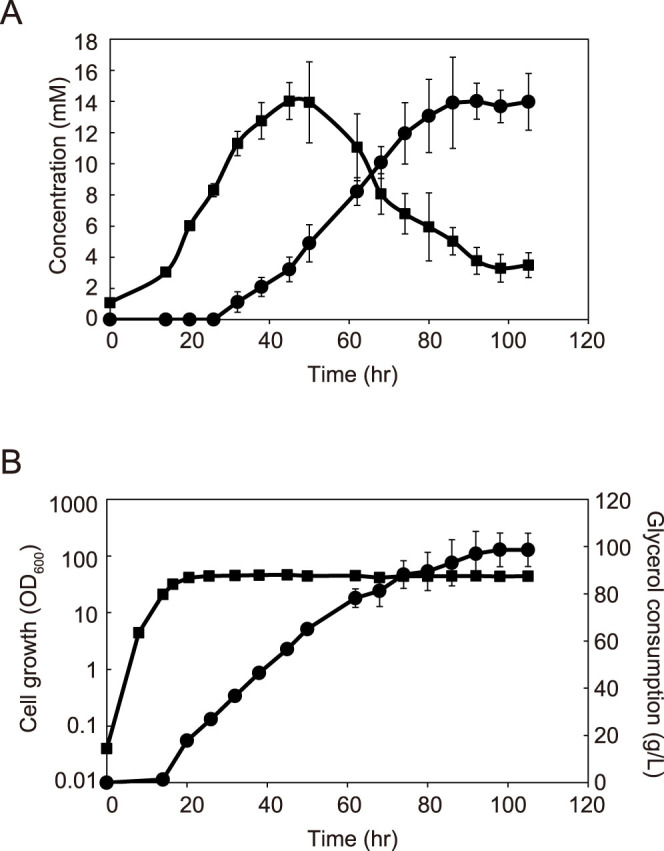
Time course of fermentative production of dopamine. (A) Concentration of dopamine (circles) and L-tyrosine (squares). (B) Glycerol consumption (circles) and cell growth (squares). Error bars indicate the standard deviation from three independent experiments.

In general, glycerol is suitable as a carbon source for material production via the shikimate pathway of *E. coli*^3^,^18^,^19^, and we thus attempted dopamine production from glycerol using a dopamine producing strain (AN1126) in a jar-fermenter. Tyrosine was produced even before induction and accumulated up to 14 mM (2.53 g/L) at 40 hours after inoculation (Fig. 2A), indicating that the tyrosine hydroxylase activity of tyrosinase was lower than the tyrosine productivity at an early stage of fermentation. After 40 hours of production, the amount of tyrosine began to decrease, presumably, because it was converted to dopamine via L-DOPA. Dopamine production started from 20 hours and continued to 90 hours after inoculation. Significant accumulation of L-DOPA was not detected during dopamine production. Approximately 14 mM (2.15 g/L, Fig. 2A) of dopamine was produced from 978 mM glycerol (90 g/L, Fig. 2B) with a production efficiency of 3.8% (note that three molecules of dopamine are synthesized from eight glycerol molecules).

### Conversion of fermentative produced dopamine to (*R,S*)-THP

**Figure 3:**
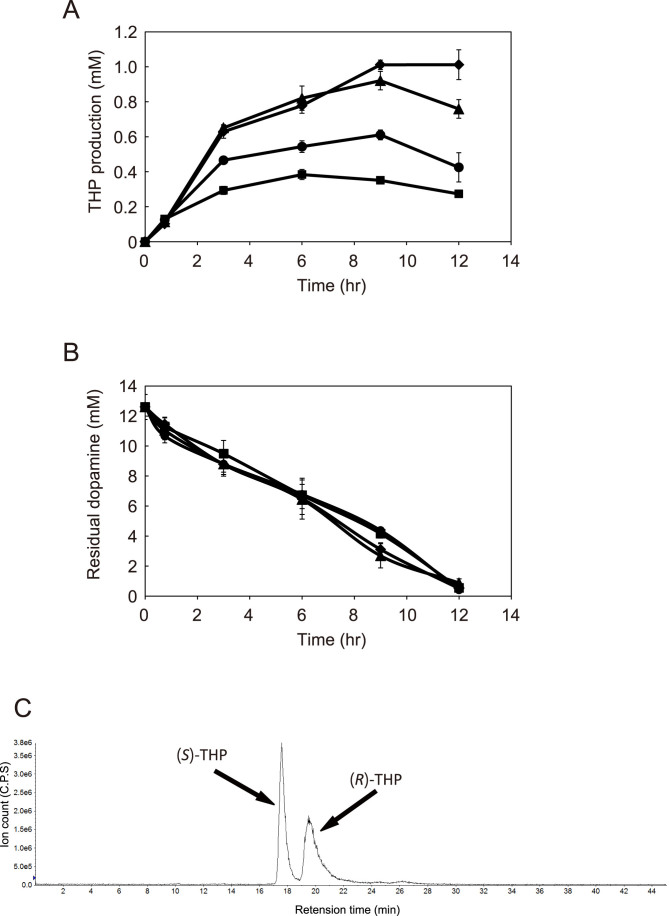
Effect of ascorbic acid on THP production and chirality of (*R,S*)-THP. THP production was performed with 3 mM (circles), 10 mM (triangles) and 30 mM (diamonds) ascorbic acid or without ascorbic acid (squares). THP production (A) and dopamine consumption (B) are represented. Error bars indicate the standard deviation from three independent experiments. (C) Chirality of (*R*,*S*)-THP was analyzed by LC-MS using a chiral column.

Dopamine containing supernatant was harvested by centrifugation so that cells containing tyrosinase did not contaminate the synthetic reaction of (*R*,*S*)-THP. (*R*,*S*)-THP production was conducted by mixing the produced dopamine with the culture of the MAO expression strain (AN1055). (*R*,*S*)-THP was continuously accumulated until 6 hours, and then sequentially degraded. The maximum yield was about 383 μM (110 mg/L) (Fig. 3A), and chirality analysis confirmed that both the R- and S-form of THP were produced (Fig. 3C). Because its four hydroxyl groups make THP easily oxidized even in the absence of tyrosinase, we speculated that larger yields could be obtained by inhibiting the autoxidation of (*R*,*S*)-THP. Ascorbic acid is a cheap antioxidant, and is used in cosmetics and foods as an additive. We therefore evaluated the antioxidant effect of ascorbic acid on (*R*,*S*)-THP production. As expected, the productivity of (*R*,*S*)-THP was improved as the ascorbic acid concentration increased (Fig. 3A). When 30 mM of ascorbic acid was added, the yield was approximately 1.0 mM (287 mg/L) which was a 2.6-fold improvement (Fig. 3A). This amount was 33-fold better compared with a previous THP production system using *A. niger*^16^. (*R*,*S*)-THP production had almost stopped at 9 hours even in the presence of 30 mM ascorbate, nevertheless, more than 3 mM dopamine remained (Fig. 3B), indicating that MAO activity was reduced after 9 hours. Dopamine continued to decrease, even after (*R*,*S*)-THP production was arrested. These data suggested that dopamine was intrinsically unstable in the production culture.

At least two kinds of by-products were detected in the (*R*,*S*)-THP production culture (Fig. S2A). From the MS/MS analysis, we could presume that one was 1-benzyl-1,2,3,4-tetrahydroisoquinoline-6,7-diol (m/z = 256) and the other was norcoclaurine (m/z = 272). The maximum amounts of 1-benzyl-1,2,3,4-tetrahydroisoquinoline-6,7-diol and norcoclaurine were equivalent to 0.41 ± 0.01 and 0.57 ± 0.04 mM THP, respectively (as calculated from the LC-MS peak areas).

The conversion efficiency of (*R*,*S*)-THP from dopamine was only 15.9% (note that one molecule of THP is synthesized from two dopamine molecules). This low efficiency was probably as a result of the instability of substrates and the production of by-products.

### Reticuline production from (*R,S*)-THP produced in this system

**Figure 4:**
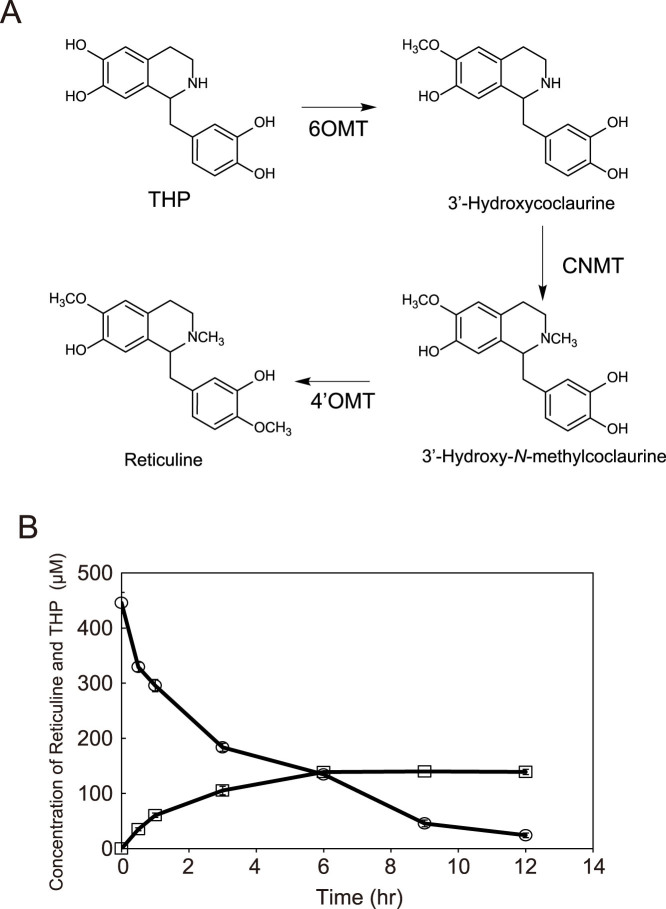
Reticuline production from (*R*,*S*)-THP synthesized by the stepwise fermentation system. (A) Reticuline synthetic pathway from THP was constructed in *E. coli* (HM66). All three methyltransferases originated from *Coptis japonica*^1^. 6OMT, 6-*O*-methyltransferase; CNMT, coclaurine *N*-methyltransferase; 4′OMT, 4′-*O*-methyltransferase. (B) Reticuline production (squares) and THP consumption (circles) were monitored. Error bars indicate the standard deviation from three independent experiments.

Reticuline is synthesized from THP through a three-step methylation process (Fig. 4A), and is an important intermediate of BIA. In fact, it is frequently the target compound of BIA production using microbes^1^,^2^,^3^,^20^,^21^. To evaluate the usability of (*R*,*S*)-THP produced by the stepwise fermentation system, we attempted to produce reticuline from (*R*,*S*)-THP by using a three-methyltransferase expression strain (HM66). Reticuline production was conducted by mixing the culture of HM66 with the same volume of (*R*,*S*)-THP containing supernatant. A total of 146 μM (48.0 mg/L) of reticuline was produced from 445 μM of (*R*,*S*)-THP, with a conversion efficiency of 32.8%, which was superior to previously reported yeast systems^2^,^4^ (Fig. 4B). Thus, these data confirmed that (*R*,*S*)-THP produced by using the stepwise fermentation system was suitable for BIA production using a microbial system.

## Discussion

The bottleneck of (*R*,*S*)-THP production is that it is easily oxidized. We found that tyrosinase has strong oxidation activity for THP, which coincides with the observation that the cytotoxicity of THP was reduced by tyrosinase in human melanocytes^22^. In order to avoid THP oxidation by tyrosinase, the synthesis of (*R*,*S*)-THP was divided into two steps. Consequently, (*R*,*S*)-THP was successfully produced using *E. coli*. In addition to avoiding undesirable reactions, multi-step culture is effective in cases where a compound produced in the downstream portion of the pathway inhibits the activity of an enzyme used upstream. Hydrogen peroxide easily oxidizes 3-deoxy-D-arabino-heptulosonate-7-phosphate synthase (AroG)^23^, which is the first enzyme of the shikimate pathway and was over-expressed for the tyrosine over-production in the BIA production pathway (Fig. 1). Hydrogen peroxide was produced during the conversion of dopamine to 3,4-DHPAA by MAO. Therefore, using a two-step culture should help to avoid the deactivation of AroG by hydrogen peroxide.

Ascorbic acid was added to the second culture for stabilization of (*R*,*S*)-THP, and improved productivity (Fig. 3A). Because dopamine also has a catechol moiety, we speculated that addition of ascorbic acid would also improve dopamine production. However, it had the opposite effect on dopamine production (Fig. S3). Tyrosine was increased in the culture containing ascorbic acid. Therefore, the decreased productivity of dopamine was likely a result of inhibition of the tyrosine hydroxylase activity of tyrosinase. In fact, it has been reported that tyrosinase is inhibited by ascorbic acid^24^. Because the ascorbic acid prevented the production of dopamine, ascorbic acid could not be used in the THP production system employing a single strain. In addition to the advantages described above, the multi-step culture allows optimization of the production conditions at each step.

In stepwise culture, the substrate produced by the prior step has to permeate through the cell membrane of the strain used in the next step. However, generally the substrate becomes diluted in the culture medium of the prior step lowering the efficiency of the next step. Dopamine was unstable in the (*R*,*S*)-THP production method presented herein, thus, it needs to be quickly processed by the second strain expressing MAO. Although we do not know how *E. coli* incorporates dopamine, the permeability of dopamine should be improved.

A previously reported THP production system using *A. niger* produced approximately 0.03 mM THP from dopamine^16^. In the present system, approximately 1.0 mM (*R*,*S*)-THP was produced (Fig. 3A), a concentration 33 times higher than that produced in the *A. niger* system. This great improvement was achieved by avoiding the oxidation of THP using the stepwise culture technique.

Although some BIAs such as salutaridine^2^, sanguinarine^4^, and morphine^25^ can be produced by a yeast system, they require the specific substrates THP or thebaine. *E. coli* systems for BIA production, including the present system, do not require any specific substrate other than glycerol, which is inexpensive. From a practical viewpoint, the low cost of the substrate is an important factor for the microbial production of secondary metabolites. Various P450 enzymes can be used in the production of different kinds of BIA, for example, CYP719B to produce morphinan alkaloids^26^ and CYP80G2 to produce magnoflorine^27^. While *E. coli* is a superior system for the practical production of the BIA building block, a yeast system is better than an *E. coli* system for P450 expression. A combination of yeast and *E. coli* systems would be a much more powerful tool for BIA production, similar to our previously reported work^1^.

However, 1-benzyl-1,2,3,4-tetrahydroisoquinoline-6,7-diol and norcoclaurine were detected as by-products. One or two hydroxy groups were absent from the benzyl moiety of these products. While the benzyl moiety of THP is derived from 3,4-DHPAA, those of 1-benzyl-1,2,3,4-tetrahydroisoquinoline-6,7-diol and norcoclaurine are presumably derived from phenylacetaldehyde (PAA) and 4-hydroxyphenylacetaldehyde (4-HPAA), respectively. When (*R*,*S*)-THP was produced from the commercial pure dopamine, these by-products were not detectable (Fig. S2B), indicating that aldehydes are synthesized from the compounds contained in the first-step culture. Because MAO can oxidize not only dopamine but also phenethylamine and tyramine, these aldehydes could have been synthesized from their cognate amine via the action of MAO. Approximately 7.6 mM phenethylamine and 2.0 mM tyramine were detected in the dopamine containing supernatant. DODC from *Pseudomonas putida* is highly specific for L-DOPA compared with L-phenylalanine and L-tyrosine^28^; accordingly, the by-products might be produced from L-phenylalanine and L-tyrosine via undesirable reactions catalyzed by MAO and DODC. Presumable by-products synthetic pathways are summarized in Supplementary Fig. S2C. The purification method should be established after the by-product analysis as the presence of by-products with similar properties to THP would make purification difficult.

BIAs are classified into two types, (*S*)-reticuline derived BIAs such as protoberberine alkaloids and (*R*)-reticuline derived BIAs such as morphinan alkaloids (Fig. S1). In plants, (*S*)-reticuline is synthesized prior to (*R*)-reticuline, and (*R*)-reticuline is produced from (*S*)-reticuline via a two-step enzymatic reaction^29^ (Fig. S1). However, because these enzymes have not been identified yet, the production of both (*R*)- and (*S*)-THP as precursors of (*R*,*S*)-reticuline is important for the synthesis of R-form derived BIAs by microbial systems, even if the S-form is still present. In the BIA production system previously constructed, the S-form specific enzyme, norcoclaurine synthetase (NCS), was used to catalyze the condensation reaction of THP synthesis^1^,^3^,^21^. Herein, the Pictet-Spengler reaction was used in the stepwise fermentation system, thus both (*R*)- and (*S*)-THP were successfully produced (Fig. 3C). (*R*,*S*)-Reticuline has been successfully produced from (*R*,*S*)-THP by microbial production^2^,^3^, and all enzymes involved in the synthesis of morphine from (*R*)-reticuline have already been identified, implying that (*R*,*S*)-THP production by this system would enable the microbial production of morphinan alkaloids such as thebaine, codeine and morphine without addition of any specific substrates.

The stepwise culture method avoids undesirable reactions and inhibition; furthermore, it allows each culture step to be optimized. At present, some plant secondary metabolites are produced by microbial systems, and various secondary metabolites may be synthesized by engineered bacteria in the future. To produce plant secondary metabolites using microbes, many enzymes need to be expressed in the microbial system. Undesirable reactions and enzyme inhibition limit efficient production. A stepwise culture method is one strategy to solve these problems, similar to multi-step synthesis in traditional chemical reactions. Indeed, although (*R*,*S*)-THP could not be produced by a single strain, it can now be produced via a stepwise culture. Producing (*R*,*S*)-THP via stepwise fermentation described herein is a simple method that does not require expensive substrates. Thus, it has potential applications in BIA production and THP-related research.

## Methods

### Plasmids and bacterial strains used in this study

Plasmids and bacterial strains are listed in Table 2.

### (*R,S*)-THP production using a single strain

Overnight culture of AN1428 was inoculated into 50 mL Terrific Broth (per liter: 12 g Tripton (Diffco), 24 g yeast extract (Diffco), 9.4 g K_2_HPO_4_ and 2.2 g KH_2_PO_4_) containing 50 mg/L ampicillin, 25 mg/L kanamycin, 2% glycerol, and 0.1 mM CuSO_4_ in a 300-mL baffled shake flask, and the culture was grown at 25 °C. IPTG (final, 0.1 mM) was added for induction 12 hours after inoculation. Samples were harvested at 6, 12, 24, and 36 hours after induction.

### Purification of His-tagged tyrosinase

The tyrosinase gene from *Ralstonia solanacealum* (*RsTYR*) with a T7 promoter was amplified by PCR using pET23a-*RsTYR*^3^ with the primer set 5′-CCCGAGCTCGATCCCGCGAAATTAATACGA-3′ and 5′-TTACTCGAGGATAACCGCAACTTCAATGG-3′. This fragment was cloned into the BglII - XhoI site of pET23a and fused to a hexahistidine tag at the C-terminus generating pET23a-*RsTYR-His* (pAN443). RsTYR-His was expressed in BL21(DE3) (Novagen, AN447). Overnight culture was inoculated into 50 mL Terrific Broth with 50 mg/L ampicillin in a baffled shake flask at 25°C. Isopropyl-β-D-thiogalactopyranoside (IPTG) (final, 1 mM) was added for induction at 12 hours after inoculation. Following further incubation for 12 hours, cells were harvested and suspended in 50 mM sodium phosphate buffer (pH 7.0) with 10% glycerol. The cell-free extract obtained by sonication was applied on a Ni-nitrilotriacetic acid affinity column (Qiagen), and eluted fractions were collected. The purity of tyrosinase was confirmed by SDS-PAGE. The most pure fraction was further purified by Amicon Ultra-15 (30 K, Millipore) treatment to eliminate the smaller proteins. The protein concentration was measured using the BCA Protein Assay Kit (Pierce). The Km value of RsTYR-His versus L-tyrosine was 1.63 ± 0.29 mM (four independent experiments), which was almost identical with that for the native RsTYR in a previous report (1.32 mM)^17^.

### Measurement of the o-diphenolase activity of tyrosinase

The *o*-diphenolase activity for L-Dopa, dopamine and (*R*,*S*)-THP were determined by decreasing the ratio of substrates. The reaction mixture (0.1 mL) contained 100 mM potassium phosphate buffer (pH 5.5), 0.01 mM CuSO_4_, 1 mM substrate and 3.48 μg protein. The reaction was stopped by addition of 2% TCA at 5, 15, and 30 minutes.

### Fermentative production of dopamine

The dopamine production strain (AN1126, Table 1) was cultured overnight in LB medium at 37°C, and 10 mL cell culture was inoculated into 1 L Terrific Broth with 5 g/L glycerol, 50 mg/L ampicillin, and 25 mg/L kanamycin. The pH, temperature (25°C constant), dissolved oxygen level, and feeding rate of glycerol were controlled as described previously^3^. Induction was carried out by adding 0.1 mM (final concentration) IPTG and 0.1 mM (final concentration) CuSO_4_ at 14 hours after inoculation (OD_600_ was approximately 20, and an OD_600_ of 1.0 was equivalent to a dry cell weight of 0.40 ± 0.01 g/L). The glycerol concentration was measured with a glycerol assay kit (Cayman Chemical) as previously described^3^. Because glycerol inhibited THP production in the second step of the culture (Fig. S4), the culture was harvested at 105 hours after inoculation, when glycerol had been completely consumed. The supernatant collected after centrifugation contained 12.6 mM dopamine. The supernatant was stored at −80°C until further use.

### Conversion of fermentative produced dopamine to (*R,S*)-THP

An overnight culture of the MAO expression strain (AN1055) was diluted 100-fold into 50 mL Terrific Broth containing 50 mg/L ampicillin in a 300-mL baffled shake flask, and the culture was grown at 25°C. IPTG (final, 1 mM) was added for induction at 12 hours after inoculation. To avoid dilution of dopamine, the cells were precipitated at 12 hours after induction when the OD_600_ was between 23 and 25, and mixed with the same volume of dopamine-containing supernatant harvested from the dopamine production culture. Because we had previously determined that BIA production required acidic conditions^21^, 100 mM 2-(*N*-morpholino) ethanesulfonic acid was added to the culture, resulting in a pH of approximately 6.0. Ascorbic acid was also added if necessary. When (*R*,*S*)-THP was produced from commercial dopamine (Fig. S2B), culture and induction were carried out using the same conditions described above. Instead of mixing the dopamine containing supernatant, 10 mM dopamine was added to the culture of AN1055. Both (*R*,*S*)-THP production methods were performed at 29°C.

### Chirality analysis

The stereoselectivity of THP was analyzed by LC–MS after separation on an Agilent HPLC system. HPLC conditions were as follows: column, CHIRALPAK IA (Daicel Chemical Industries); solvent system, formate solution (pH 2.0): methanol (83:17); flow rate, 0.3 mL/min at 40°C. Identification of the R- and S-forms was conducted by comparing with the THP produced in vitro using NCS (S-form specific enzyme), which contained a larger amount of (*S*)-THP than (*R*)-THP. THP was confirmed by its select ion (m/z = 288) and MS/MS fragment pattern (daughter ions: m/z = 123 and 164).

### Reticuline production

The supernatant of the (*R*,*S*)-THP production culture was harvested by centrifugation, and found to contain 990 μM (*R*,*S*)-THP. The three-methyltransferase expression strain (HM66) was cultured in Terrific Broth containing 50 mg/L chloramphenicol for 12 hours. After induction was carried out by the addition of 1 mM (final concentration) IPTG, the culture was continued for more 12 hours. The (*R*,*S*)-THP containing supernatant was added to the same volume of HM66 culture and 1% glucose. The production mixture was incubated at 25°C.

### Detection and quantification of chemical compounds

For the in vitro assay, the samples were analyzed by HPLC using a Discovery HS F5 column (Supelco) after centrifugation. Compounds were separated on the column by using 30% acetonitrile in 10 mM ammonium formate (pH 3.0) at a flow rate of 0.5 mL/min. L-DOPA, dopamine, and THP were monitored by measuring the absorbance at 280 nm. The samples collected from the production culture were treated with 2% trichloroacetic acid and centrifuged. L-tyrosine, L-DOPA, and dopamine were analyzed by HPLC using the same conditions as the in vitro assay, except for the mobile phase where the concentration of acetonitrile was increased from 3 to 20% in 10 mM ammonium formate (pH 3.0). THP (m/z = 288), reticuline (m/z = 330), 1-benzyl-1,2,3,4-tetrahydroisoquinoline-6,7-diol (m/z = 256), norcoclaurine (m/z = 272) and phenethylamine (m/z = 122) were analyzed by LC-MS using the same method as described previously^3^. Because by-products were not available commercially, their concentrations were estimated as equivalents of THP.

## Author Contributions

A.N. and H.M. conceived and designed all experiments. A.N. and C.M. performed the experiments and data analysis. E.M., T. Koyanagi, T. Katayama and K.Y. discussed the results. H.K. and F.S. supervised the project. A.N., H.M. and F.S. wrote the manuscript. All authors reviewed the manuscript.

## Supplementary Material

Supplementary Information (*R,S*)-Tetrahydropapaveroline production by stepwise fermentation using engineered Escherichia coli.

## Figures and Tables

**Table 1:** *o*-Diphenolase activity of tyrosinase

*o*-Diphenolase activity (nmoles/min/mg protein)		
L-DOPA	Dopamine	THP
315 ± 32	57 ± 11	216 ± 36

Values represent mean ± SEM from three independent experiments.

**Table 2:** Plasmids and bacterial strains

	Genotype	Description	Reference
*Plasmids*			
pAN23	pCOLADuet-1-*tyrA^fbr^*-*aroG^fbr^*-*tktA*-*ppsA*	Gene set for L-tyrosine over-production	3
pAN349	pET23a-*RsTYR*-*DODC*	Gene set for conversion of L-tyrosine to dopamine	3
pAN443	pET23a-*RsTYR*-His	For purification of tyrosinase with hexahistidine tag	This study
pAN465	pGS21a-MAO	Monoamine oxidase for conversion of dopamine to (*R*,*S*)-THP supplied from Genscript	3
pAN828	pET23a-*RsTYR*-*DODC*-*MAO*	Used for (*R*,*S*)-THP production by a single strain	This study
pHM65	pACYC184-*6OMT*-*4′OMT*-*CNMT*	Gene set for conversion of THP to reticuline	1
*Strains*			
BL21(DE3)	F^−^ *ompT* *hsdSB*(rB^−^, mB^−^) *gal* *dcm* (DE3)		Novagen
AN447	BL21(DE3) harboring pAN443	His-tagged tyrosinase expression strain	This study
AN1055	BL21(DE3) harboring pAN465	MAO expression strain	This study
AN1126	BL21(DE3) *tyrR* null harboring pAN23 and pAN349	Dopamine producer	3
AN1428	BL21(DE3) *tyrR* null harboring pAN23 and pAN828	(*R*,*S*)-THP production in a single strain	This study
HM66	BL21(DE3) harboring pHM65	Reticuline producer from THP	1
