# Supplementary material for: (R,S)-Tetrahydropapaveroline production by stepwise fermentation using engineered Escherichia coli
Source: Sci Rep. 2014 Oct 21;4:6695. doi: 10.1038/srep06695 (PMC4204043; doi:10.1038/srep06695)
Supplement: Supplementary Information — (R,S)-Tetrahydropapaveroline production by stepwise fermentation using engineered Escherichia coli. [file srep06695-s1.pdf]

Supplementary information

(*R*,*S*)-Tetrahydropapaveroline production by stepwise fermentation using engineered *Escherichia coli*.

Akira Nakagawa[1], Chiaki Matsuzaki[1], Eitaro Matsumura[1], Takashi Koyanagi[1], Takane Katayama[1], Kenji Yamamoto[1], Fumihiko Sato[2], Hidehiko Kumagai[1], and Hiromichi Minami[1]*

*Correspondence and requests for materials should be addressed to H.M. (minami@ishikawa-pu.ac.jp)

[1]Research Institute for Bioresources and Biotechnology, Ishikawa Prefectural University, Nonoichi-shi, Ishikawa 921-8836, Japan.
[2]Division of Integrated Life Science, Graduate School of Biostudies, Kyoto University, Oiwake-cho, Kitashirakawa, Sakyo-ku, Kyoto 606-8502, Japan

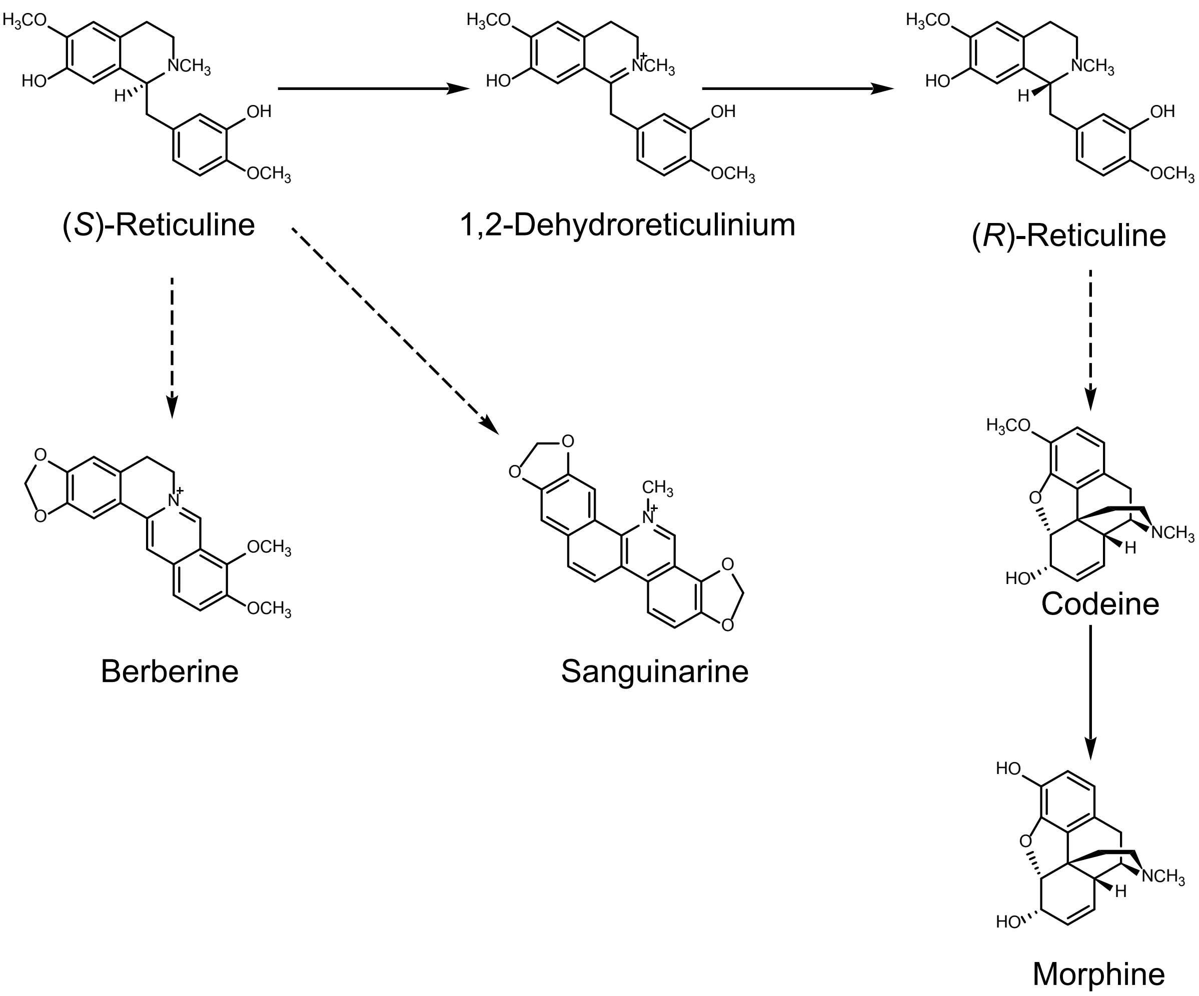

Supplementary Figure S1: (*R*)-reticuline conversion from (*S*)-reticuline and examples of reticuline derived pharmaceuticals.

(*R*)-reticuline is converted from (*R*)-reticuline via 1,2-Dihydroreticulinium by two unknown enzymes. Dash arrows indicate multistep reactions.

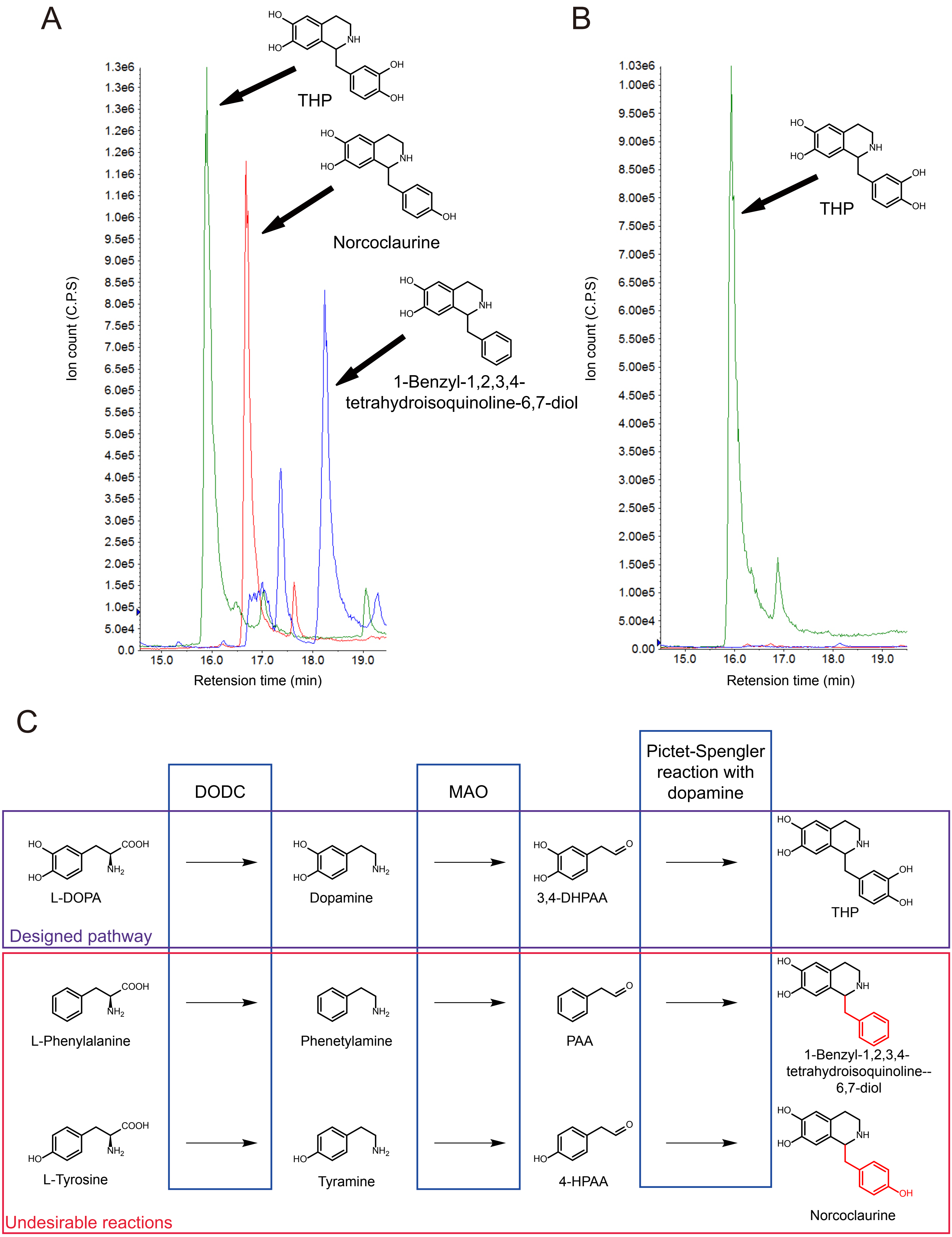

Supplementary Figure S2: By-products analysis of (*R*,*S*)-THP production culture.

Supplementary Figure S2: By-products analysis of (*R*,*S*)-THP production culture.

(A,B)LC-MS analysis of (*R*,*S*)-THP production culture in select ion mode. (*R*,*S*)-THP was obtained from the fermentative produced dopamine (A) or the pure commercial dopamine (B). Green, m/z=288; red, m/z=272; blue, m/z=256. (C) Designed THP production pathway (purple blanket) and presumable by-product synthetic pathway (red blanket). Amino acids are converted to cognate amines by DODC. Aldehydes derived from amines are synthesized by MAO. Finally, each aldehyde is united with dopamine via a spontaneous Pictet-Spengler reaction, producing THP, 1-benzyl-1,2,3,4-tetrahydroisoquinoline-6,7-diol or norcoclaurine.

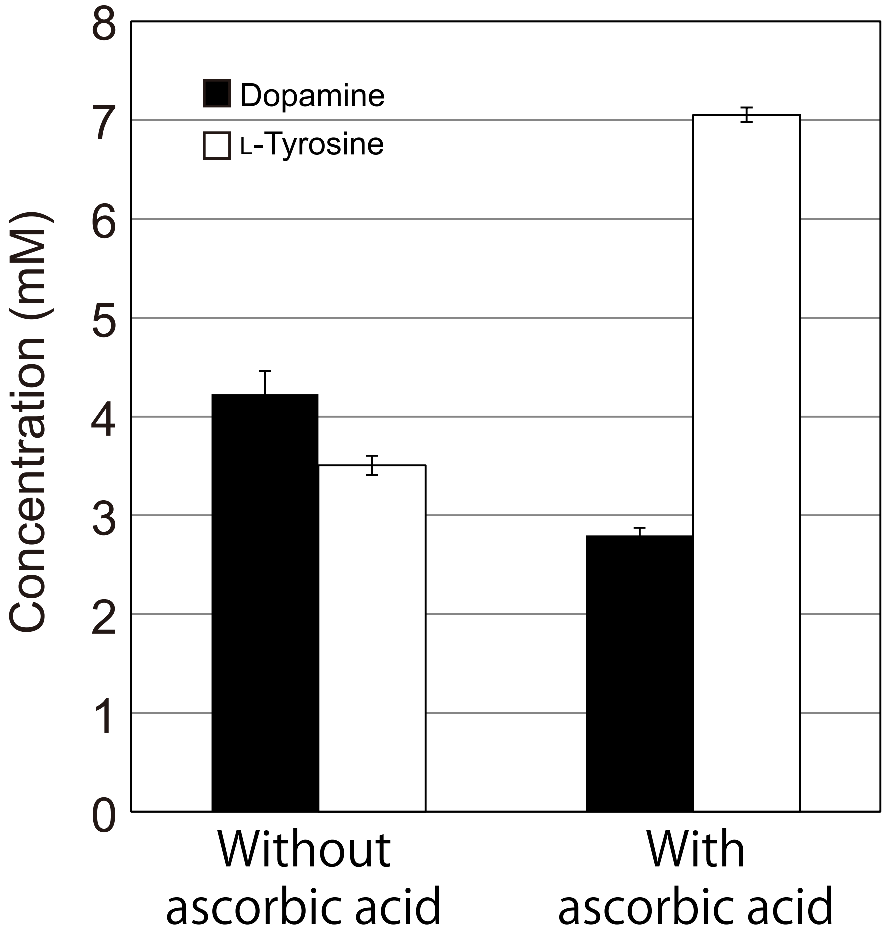

Supplementary Figure S3: The effect of ascorbic acid on the dopamine production.

The dopamine production strain (AN1126) was cultured overnight in LB medium at 37°C, and was inoculated into 50 mL of Terrific Broth with 2% glycerol, 50 mg/L ampicillin and 25 mg/L kanamycin in a 300 mL of baffled-flask. Induction was carried out by addition of 0.1 mM IPTG and 0.1 mM $CuSO_4$ with or without 30 mM ascorbic acid at 14 hours after inoculation. Samples were harvested at 36 hours after inoculation. The concentrations of dopamine (black bars) and tyrosine (white bars) were analyzed by HPLC. Error bars indicate the standard deviation from three independent experiments.

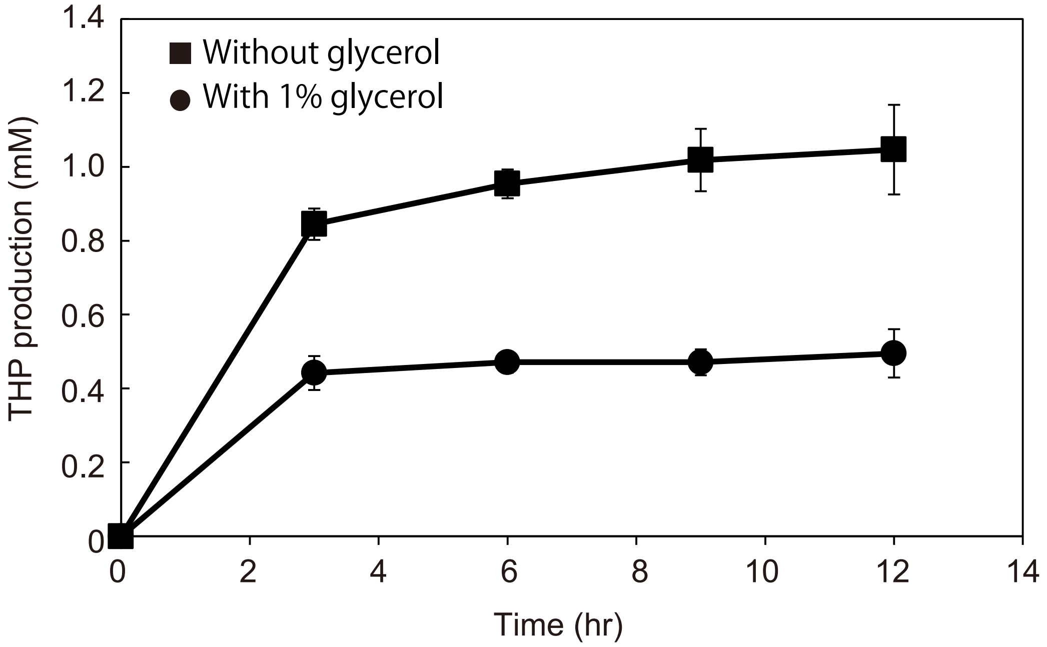

Supplementary Figure S4: Inhibitory effect on THP production in a second step culture.
THP production from dopamine containing supernatant was performed with (circles) or without 1% glycerol (squares). AN1055 (MAO expression strain) cells were harvested and suspended by dopamine containing supernatant with 30 mM ascorbic acid. When 1% glycerol was added, THP production was inhibited. Therefore, for THP production, we used dopamine containing supernatant which does not include residual glycerol in the first step culture.
